# Multiple detection of both attractants and repellents by the dCache‐chemoreceptor SO_1056 of *Shewanella oneidensis*

**DOI:** 10.1111/febs.16548

**Published:** 2022-06-24

**Authors:** Anne Boyeldieu, Jean‐Pierre Poli, Amine Ali Chaouche, Henri‐Pierre Fierobe, Marie‐Thérèse Giudici‐Orticoni, Vincent Méjean, Cécile Jourlin‐Castelli

**Affiliations:** ^1^ Laboratoire de Bioénergétique et Ingénierie des Protéines (BIP, UMR7281), Centre National de la Recherche Scientifique, Institut de Microbiologie de la Méditerranée (IMM), Institut Microbiologie, Bioénergies et Biotechnologie (IM2B) Aix Marseille Université France; ^2^ Université de Corse Pasquale Paoli Corte France; ^3^ Laboratoire de Chimie Bactérienne (LCB, UMR7283), Centre National de la Recherche Scientifique, Institut de Microbiologie de la Méditerranée (IMM), Institut Microbiologie, Bioénergies et Biotechnologie (IM2B) Aix Marseille Université France; ^4^ Present address: Laboratoire de Microbiologie et de Génétique Moléculaires, UMR5100, Centre de Biologie Intégrative (CBI), Centre National de la Recherche Scientifique (CNRS) Université de Toulouse, UPS France

**Keywords:** bacterial chemotaxis, chemoreceptors, dCache domain, isothermal titration calorimetry, ligand binding, thermal shift assay

## Abstract

Chemoreceptors are usually transmembrane proteins dedicated to the detection of compound gradients or signals in the surroundings of a bacterium. After detection, they modulate the activation of CheA‐CheY, the core of the chemotactic pathway, to allow cells to move upwards or downwards depending on whether the signal is an attractant or a repellent, respectively. Environmental bacteria such as *Shewanella oneidensis* harbour dozens of chemoreceptors or MCPs (methyl‐accepting chemotaxis proteins). A recent study revealed that MCP SO_1056 of *S. oneidensis* binds chromate. Here, we show that this MCP also detects an additional attractant (l‐malate) and two repellents (nickel and cobalt). The experiments were performed *in vivo* by the agarose‐in‐plug technique after overproducing MCP SO_1056 and *in vitro*, when possible, by submitting the purified ligand‐binding domain (LBD) of SO_1056 to a thermal shift assay (TSA) coupled to isothermal titration calorimetry (ITC). ITC assays revealed a K_*D*_ of 3.4 μm for l‐malate and of 47.7 μm for nickel. We conclude that MCP SO_1056 binds attractants and repellents of unrelated composition. The LBD of SO_1056 belongs to the double Cache_1 family and is highly homologous to PctA, a chemoreceptor from *Pseudomonas aeruginosa* that detects several amino acids. Therefore, LBDs of the same family can bind diverse compounds, confirming that experimental approaches are required to define accurate LBD‐binding molecules or signals.

## Graphical abstract

Chemoreceptors or MCPs (methyl‐accepting chemotaxis proteins) detect signals and modulate the activation of CheA‐CheY, the core of the chemotactic pathway, to allow cells to orient themselves in their surroundings. The chemoreceptor SO_1056 of *Shewanella oneidensis* is involved in chemotaxis towards attractants (chromate and l‐malic acid) and repellents (nickel and cobalt). The purified Ligand‐Binding domain of SO_1056 (LBD1056) proved to bind directly l‐malic acid and nickel *in vitro*.

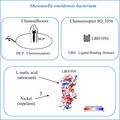

**Abbreviations**
4HB: 4‐helix bundle
DAHL: double all‐helical ligand‐binding domain
HBM: helical bi‐modular
ITC: isothermal titration calorimetry
LBD: ligand‐binding domain
MCPs: methyl‐accepting chemotaxis proteins
PBP: periplasmic binding protein
TSA: thermal shift assay

## Introduction

Methyl‐accepting chemotaxis proteins (MCPs), also called chemoreceptors, are crucial for bacteria to move and orient themselves in their environment. By detecting signals and transducing them to the core CheA/CheY chemotaxis system, they impact flagellum motor rotation allowing bacteria to navigate in gradients of various molecules [1, 2, 3]. These latter called chemoeffectors can be either attractants or repellents, provoking bacterial movement upwards or downwards a gradient of concentration, respectively.

A common feature of chemoreceptors is the presence of a conserved cytoplasmic signalling domain (annotated as MA domain or MCPsignal domain in the SMART and Pfam databases, respectively). This characteristic domain is involved in the transduction of signals to the CheA histidine kinase. Chemoreceptors are divergent in size and in cellular localization, although most of them are anchored to the membrane. They can be classified, according to the number of helical heptads (repeated pattern of seven residues) contained in their signalling domain, into diverse families (64H, 58H, 52H, 48H, 44H, 42H, 40H, 38H, 36H, 34H, 28H and 24H) [4]. They have also been classified according to the size of their ligand‐binding domain (LBD) into two clusters: cluster I (average length 156 amino acids) and cluster II (average length 262 amino acids) [5]. Chemoreceptors belonging to cluster I contain mono‐modular LBDs such as 4HB (4‐helix bundle) and sCache (single Cache), while those of cluster II present bi‐modular LBDs such as dCache (double Cache) and HBM (helical bi‐modular) [3, 5].

Chemoreceptor‐encoding genes are easily identified based on high conservation of the encoded signalling domains, and a large number of them have been predicted from bacterial genomes [6]. However, the ligand(s) detected by most of the chemoreceptors are still unknown. Bioinformatics approaches allow annotation of the LBD, but this is not sufficient to assign a ligand to an MCP. Indeed, the same ligand can be recognized by chemoreceptors containing different types of LBD. Structure‐based homology searches can sometimes give a hint of the type of ligand an MCP can bind, but even if that is the case, experiments should then be performed to confirm ligand binding [7]. Experimental data are, therefore, necessary to increase our knowledge of signal detection by chemoreceptors. Various approaches are used to couple signal(s) to MCP(s), such as *in vivo* monitoring of the chemotactic behaviour of *mcp*‐deleted or *mcp*‐overexpressing strains, and *in vitro* binding assays using purified LBD recombinant proteins (including thermal shift assay, differential scanning fluorimetry and isothermal titration calorimetry) [3, 8, 9, 10, 11].

The number of chemoreceptors has been suggested to be related to the habitat and the lifestyle of bacteria [5, 12]. Those living in stable or restricted niches possess few MCPs, as is the case for *Escherichia coli* that contains only five MCPs. Other bacteria encountering stressful and/or highly variable environments use a higher number of chemoreceptors to adapt their behaviours according to the various signals they must detect for survival and growth. *Shewanella oneidensis*, a Gram‐negative proteobacterium isolated from an aquatic environment, possesses 27 chemoreceptors. This bacterium withstands variations of temperature, pH, salinity and pressure in its environment and is able to use several electron acceptors including metals for its respiration [13, 14].

*Shewanella oneidensis* has been shown to be chemotactic, a behaviour which is controlled by the Che3 chemosensory system [15, 16, 17]. The other functional chemosensory system (Che1) is involved in the control of RpoS activity, while the Che2 system is probably not functional due to the presence of insertion sequence into two Che2 genes [18]. *S. oneidensis* is attracted by various respiratory compounds (TMAO, DMSO, nitrate, nitrite, fumarate, iron and manganese) but also by l‐malate and more surprisingly by chromate, while it is repelled by nickel and cobalt [16, 19, 20]. The chemotactic behaviour in response to the respiratory compounds results from an energy taxis mechanism. Indeed, these compounds are not detected *per se*, but they need to be used by a functional respiratory system for taxis to occur. Five chemoreceptors, one major (SO_2240) and four minor (SO_3282, SO_3642, SO_3890 and SO_4454), have been proved to be involved in this process [16]. Interestingly, the SO_2240 chemoreceptor has also been shown to be involved in a bacterial behaviour called congregation, resulting in the accumulation of cells around insoluble electron acceptors [21, 22]. Although this MCP presents an LBD with an sCache domain, the mechanism by which it triggers energy taxis is still unknown.

Recently, we investigated how chromate, a toxic metal recognized as an attractant by *S. oneidensis*, could be detected. Seven Cache‐containing chemoreceptors were tested for their involvement in chromate attraction. Among them, two allowed an enhanced response towards chromate when overproduced (SO_0987 and SO_1056) but only SO_1056 was able to detect chromate by direct binding to its LBD [8]. This latter chemoreceptor was the focus of this study. Using *in vivo* and *in vitro* approaches, we demonstrated that this MCP is also involved in attraction towards l‐malate and repulsion to nickel and cobalt and that it directly binds l‐malate and nickel via its dCache‐containing LBD.

## Results

### Overexpression of the MCP‐encoding *so_1056* gene triggers an enhanced response towards l‐malate, nickel and cobalt

We have previously shown that the chemoreceptor SO_1056, which belongs to the 40H family, is involved in the attraction of *S. oneidensis* towards chromate [8]. The LBD of SO_1056 contains a double Cache domain (dCache_1). Since these domains contain two modules, one membrane‐distal and one membrane‐proximal, both susceptible to binding a ligand, we wondered whether SO_1056 could detect other chemoeffectors in addition to chromate. Among the known chemoeffectors for *S. oneidensis*, an attractant (l‐malate) and the two repellents (nickel and cobalt) have not been attributed to any of the 27 chemoreceptors encoded in the genome. To test the putative involvement of SO_1056 in the chemotactic response towards these chemoeffectors, we overexpressed the *so_1056* gene and surveyed the chemotactic behaviour of the resulting strain using the agarose‐in‐plug bridge assay. Indeed, we recently showed that overexpression of an MCP‐encoding gene can result in an enhanced chemotactic response towards the chemoreceptor ligand [8]. We chose to perform overexpression in the strain *Shewanella massilia*, which responds to the same compounds as *S. oneidensis* and has the advantage of swimming faster than *S. oneidensis*, allowing better visualization of the repellent responses [20, 23]. To estimate the number of cells present around the chemoeffector‐containing plugs, the microscopy images were analysed and pixel intensities at various ranges from the edge of the plug were calculated.

**Fig. 1:**
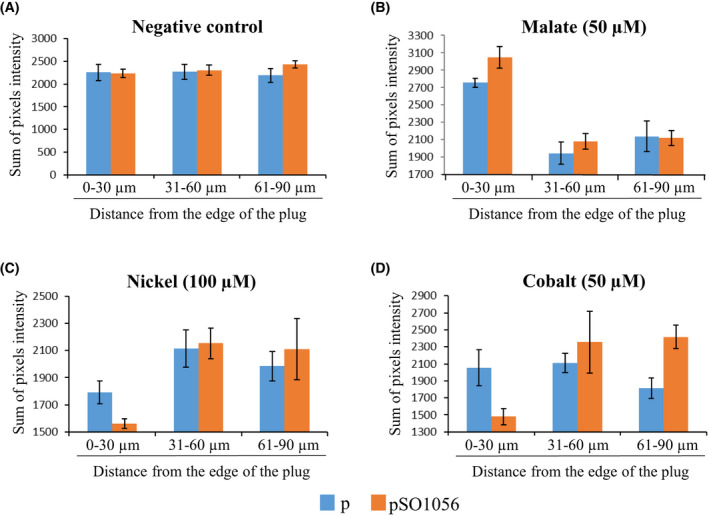
Chemotactic behaviour of *Shewanella massilia* cells containing either an empty vector (p) or the plasmid (pSO1056) bearing the MCP‐encoding *so_1056* gene towards various compounds. The chemotactic behaviours of the strains towards a plug that contained either water (negative control) (A), l‐malate (B), nickel chloride (C) or cobalt chloride (D) were tested using an agarose‐in‐plug bridge assay. Photographs were taken at the edges of the plug after 35 min of incubation. Pixel intensities of each photograph, which are proportional to the number of cells around the plugs, were obtained using imagej. Graphs represent the sum of intensities of pixels located within 30 μm ranges along a 90 μm axis starting from the edge of the plug. Error bars represent the standard deviation of the mean (*n* = 10). For graphical purposes, the *y*‐axes of the graphs were adapted to each condition.

As shown in Fig. 1A, when no effector was added to the plug (negative control), the sums of pixel intensities were similar whatever the distance from the plug, indicating that the cells are homogeneously distributed around the plug. In contrast, when l‐malate was added to the plug, a higher sum of pixel intensities was observed for the 0–30 μm range compared to the 30–60 and 60–90 μm ranges, indicating that a higher number of cells are present close to the plug and thus confirming that *S. massilia* is attracted by l‐malate (Fig. 1B). Strikingly, overexpression of the *so_1056* gene resulted in an even higher sum of pixel intensities for the 0–30 μm range. This result strongly suggests that the SO_1056 chemoreceptor is involved in the attraction towards l‐malate.

When nickel was added to the plug, the number of cells was lower at the closest range (0–30 μm) than further away, confirming that *S. massilia* is repelled by this metal. Interestingly, the number of cells close to the plug was significantly lower when the *so_1056* gene was overexpressed, indicating that repulsion is enhanced in this condition (Fig. 1C). A similar phenomenon was observed when cobalt was added to the plug, since the number of cells also decreased at the closest range in the *so_1056* overexpressing strain (Fig. 1D). This result supports involvement of the chemoreceptor SO_1056 in the chemotactic response towards the two repellents, nickel and cobalt.

Altogether, our results strongly suggest that the chemoreceptor SO_1056 plays a role in both attraction and repulsion for *S. oneidensis* [8].

### The LBD of SO_1056 directly binds l‐malate and nickel

**Fig. 2:**
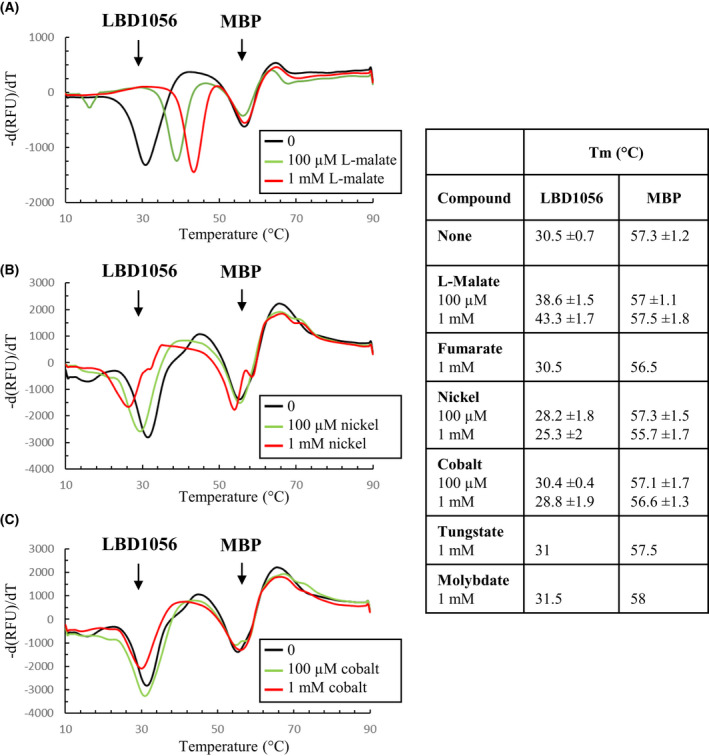
Interaction tests with the MBP‐LBD1056 protein and different compounds using thermal shift assay. The MBP‐LBD1056 protein was incubated in the presence of SYPRO Orange and various concentrations of l‐malate (A), nickel chloride (B) or cobalt chloride (C) and submitted to a temperature gradient from 10 to 90 °C. Graphs represent the first derivative of the fluorescence emission (−d(RFU)/dT, RFU: Raw Fluorescence Unit) as a function of temperature. The melting temperatures (Tm) of each peak are listed in the table on the right (mean values with standard deviation, *n* ≥ 5). The Tm obtained in the presence of control molecules (fumarate, tungstate and molybdate) is also indicated in the table (mean values with standard deviation, *n* = 2). Each graph is coloured according to the concentration of the tested ligand, as indicated. All graphs are representative of at least three independent experiments.

In order to decipher whether SO_1056 detects the identified chemoeffectors directly or not, we purified its LBD fused to maltose‐binding protein (MBP‐LBD1056) and performed thermal shift assays (TSAs). As previously observed [8], the curve of the first derivative of fluorescence emission relative to temperature revealed two peaks for the MBP‐LBD1056 protein, one related to the LBD moiety with a Tm of 30.5 °C and one related to the MBP moiety with a Tm of 57.3 °C (Fig. 2).

When the TSA was performed in the presence of 100 μm l‐malate, the Tm of the LBD moiety was increased by 8 °C, while that of the MBP moiety was unaffected (Fig. 2A). The use of 1 mm l‐malate further increased the Tm of the LBD, while that of the MBP was not modified. When fumarate, another C4‐dicarboxylic acid which is also an attractant for *S. oneidensis*, was used at 1 mm, the Tm of LBD1056 was not modified, indicating that fumarate does not interact with LBD1056 and that the effect of l‐malate is thus specific. These results indicate that the LBD of the chemoreceptor SO_1056 directly binds l‐malate, in addition to chromate [8].

When the MBP‐LBD1056 protein was incubated in the presence of nickel chloride, a shift of the LBD1056 peak was observed. In contrast to what happened in the presence of the l‐malate attractant, the Tm of the LBD was decreased in the presence of nickel, while the Tm of the MBP was poorly affected (Fig. 2B). Indeed, we measured a Tm for the LBD of 28.2 °C in the presence of 100 μm nickel chloride and of 25.3 °C in the presence of 1 mm nickel chloride, a decrease of 2.3 and 5.2 °C, respectively compared with the experiment without nickel. As a control, two other transition metals (molybdate and tungstate) were tested at 1 mm. No significant effect on the curve of the MBP‐LBD1056 protein was observed, the Tm of the MBP and the LBD moieties being only slightly affected (Fig. 2). These results indicate that the LBD of the SO_1056 chemoreceptor specifically binds nickel, resulting in thermal destabilization of this domain. Interestingly, the resulting effects of chemoeffector binding to the chemoreceptor are the opposite, the attractants stabilizing the protein with regard to temperature and the repellent destabilizing it.

When cobalt chloride was used at 100 μm, the Tm of the LBD was not modified. Only a slight decrease in the Tm was observed when cobalt was added at 1 mm (Fig. 2C). Since ΔTm was below 2 °C, we considered that the effect of cobalt on the LBD was not relevant. This means either that cobalt is not directly detected by the LBD of SO_1056 or that TSA did not work in the case of cobalt for an unknown reason.

**Fig. 3:**
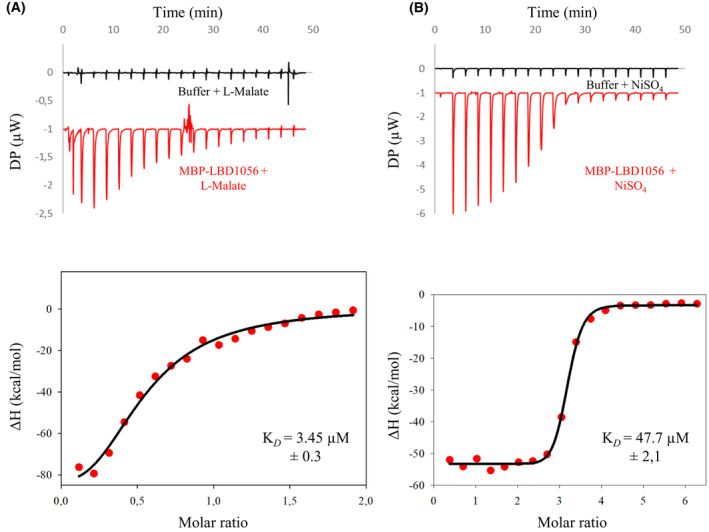
Isothermal titration calorimetry of MBP‐LBD1056 with l‐malate and nickel. Top graphics show heat exchange upon ligand titration, first with dialysis buffer (control, black line) and then with MBP‐LBD1056 (red line). Bottom graphics show the integrated data after control subtraction fitted using Hill equation. The red dots represent experimental data, and the black line represents the fit. The data shown are representative of two independent experiments. (A) represents the interaction between MBP‐LBD1056 (15 μm) and l‐malate (150 μm), while (B) shows the interaction between MBP‐LBD1056 (15 μm) and nickel sulfate (500 μm).

To further validate the direct binding of l‐malate and nickel to the LBD of SO_1056 and to quantify the binding affinity of these interactions, we performed isothermal titration calorimetry (ITC) with the purified MBP‐LBD1056 protein at 25 °C. As shown in Fig. 3, both l‐malate and nickel elicited an exothermic reaction, although the profiles were not similar. These data show an unambiguous interaction between MBP‐LBD1056 and l‐malate or nickel. Since TSA indicated that neither l‐malate nor nickel affects the thermostability of the MBP moiety, it seems reasonable to assume that the exothermic reactions are due to ligand binding to the LBD1056 moiety. The data obtained could not be fitted with the simplest 1–1 reaction model, and the apparent K_*D*_ were determined using Hill equation, suggesting a multisite interaction model. The apparent K_*D*_ for l‐malate and nickel were, respectively, 3.4 μm ± 0.3 and 47.7 μm ± 2.1.

We also carried out ITC experiments using chromate and cobalt as ligands but, unfortunately, no K_*D*_ could be obtained.

Altogether, these results indicate that the chemoreceptor SO_1056 directly binds not only attractants (l‐malate and chromate) but also at least one repellent (nickel).

### The LBD of SO_1056 contains conserved residues that could be involved in ligand binding

To gain insight into how SO_1056 might bind its ligands, we searched for proteins with LBD homologous to that of SO_1056 using Smart BLAST and the LBD1056 sequence as a query. Among the best hits, two chemoreceptors were found: PctA from *Pseudomonas aeruginosa* and McpB from *Bacillus subtilis* with *E*‐value of 5e^−25^ and 4e^−13^, respectively. These two chemoreceptors contain a double Cache domain (dCache_1 domain for PctA and dCache_2 for McpB), as is also the case for SO_1056 (dCache_1). PctA is a broad‐range amino acid chemoreceptor, and structures of its LBD were obtained in complex with different l‐amino acids [24].

**Fig. 4:**
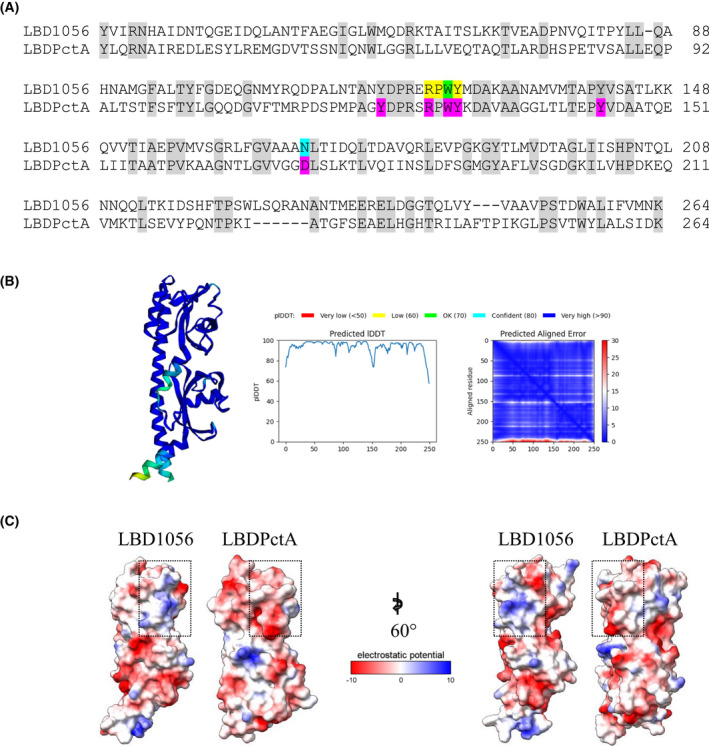
Amino acid sequence alignment and structure prediction of the LBDs of SO_1056 and PctA. (A) Amino acid sequence alignment of the LBDs of SO_1056 (LBD1056) and PctA of *Pseudomonas aeruginosa* (LBDPctA). Conserved residues between the two proteins are shaded in light grey. Conserved residues of the AA_motif of PctA are highlighted in pink. The residue of SO_1056 that has been replaced by an alanine is highlighted in green. The four‐residues sequence (RPWY) of SO_1056 that has been replaced by an LLDS sequence is highlighted in yellow. The N170 residue of SO_1056 that has been replaced by D is highlighted in blue. UniProtKB accession numbers are: Q8EHZ8 (SO_1056) and G3XD24 (PctA). The alignment was recovered from the Smart BLAST server after uploading the SO_1056 LBD sequence. (B) Cartoon representation of the structural prediction for LBD1056. The structural prediction was obtained using the software alphafold. (C) Electrostatic surface potential of the LBDs of SO_1056 (LBD1056) and PctA (LBDPctA). The electrostatic surfaces were calculated with the default coulombic command in chimerax. Regions of negative potential are coloured red, those of positive potential are coloured blue. The region of PctA, which binds its ligands, and the corresponding region in alphafold's prediction for SO_1056, are surrounded by dashed rectangles. Structures on the left are in the same orientation as in (B), while structures on the right are rotated 60°.

Alignment of SO_1056 and PctA LBDs revealed that several residues involved in PctA‐ligand coordination are conserved in LBD1056 (Fig. 4A). Interestingly, it was recently proposed that a subclass of dCache_1 domain contains a motif allowing amino acid ligand binding (called AA_motif). The consensus proposed for this motif [Y_121_ R_126_ W_128_ Y_129_ Y_144_ D_173_, numbered according to PctA, Fig. 4A] contains two parts: in the N‐terminal part, the residues Y121, R126 and W128 are involved in key contacts with the carboxyl group of the ligand, while the C‐terminal part contains Y144 and D173 making contact with the amino group of the ligand [25]. As observed in Fig. 4A, the LBD of SO_1056 presents a non‐canonical AA_motif. Indeed, the first part of the motif is conserved (Y118, R123 and W125, numbered according to SO_1056), while in the second part an N170 is found at the position corresponding to D173 in PctA. We, therefore, wondered whether LBD1056 could bind amino acids such as PctA. The purified MBP‐LBD1056 protein was submitted to TSA in the presence of three amino acids shown to co‐crystallize with PctA, namely L‐Trp, L‐Ser and L‐Met. As shown in Table 1, no significant Tm shift could be observed for the LBD moiety in the presence of these amino acids. This result strongly suggests that the LBD of SO_1056 is not involved in amino acid binding. This seems to be consistent with the work of Gumerov et al. [25] who showed that mutation of D173 to N in PctA abolished l‐Trp binding and strongly reduced l‐Ala binding.

**Table 1:** Melting temperatures for MBP‐LBD1056 and MBP‐LBD1056_N170D_ obtained using TSA in the presence of various compounds. The melting temperature (Tm) is indicated for each moiety (LBD and MBP) in the absence or presence of different compounds at various concentrations (mean values with standard deviation, *n* ≥ 3).

Protein tested	Compound		Tm (°C)	
			LBD (1056 or 1056_N170D_)	MBP
MBP‐LBD1056	None		30.8 ± 0.4	58.5
	l‐Met	100 μm	31.8 ± 0.4	58.5
		1 mm	31 ± 0.7	58.5
	l‐Ile	100 μm	31.3 ± 1.1	58.5
		1 mm	31 ± 0.7	58.5
	l‐Trp	100 μm	31 ± 0.7	58.8 ± 0.4
		1 mm	31.3 ± 0.4	58.5
	Maltose	1 mm	30 ± 2.1	62.3 ± 0.4
MBP‐LBD1056_N170D_	None		38.6 ± 0.9	56.9 ± 1.3
	l‐malate	100 μm	39.5 ± 0.7	56
		1 mm	41 ± 0.6	56.8 ± 1.2
	Nickel	100 μm	36.8 ± 0.4	56.5
		1 mm	35	56.4 ± 1.3
	l‐Met	100 μm	39.3 ± 0.4	56
		1 mm	39.3 ± 1.1	56
		4 mm	38.8 ± 0.3	57.5
	l‐Ile	100 μm	39.3 ± 0.4	55.5
		1 mm	39 ± 0.7	55.8 ± 0.4
		4 mm	38.8 ± 0.3	57.8 ± 0.3
	l‐Trp	100 μm	39 ± 0.7	55.8 ± 0.4
		1 mm	38 ± 0.7	55.5
		4 mm	38.2 ± 0.3	57.5
	Maltose	1 mm	38.7 ± 1.1	62.3 ± 0.3

As one ligand of SO_1056 (l‐malate) contains carboxyl moieties like the ligands of PctA, we wondered whether the conserved residues found in the N‐terminal part of the non‐canonical AA_motif of SO_1056 could also be involved in ligand binding. We, therefore, overproduced a mutant LBD1056, in which the RPWY sequence (position R123 to Y126) was replaced by LLDS. Indeed, mutation of the four equivalent residues in the dCache domain of the *Bacillus subtilis* kinase KinD abolished the response to l‐malic acid [26]. Moreover, this four‐residue sequence contains three of the conserved amino acids present in the N‐terminal part of the AA_motif (positions R123, W125 and Y126 in SO_1056). We also overproduced a mutated version of LBD1056, in which the residue W125 was replaced by alanine. This residue was also identified as being important for ligand binding in the *Campylobacter jejuni* dCache‐containing chemoreceptor Tlp3 [27].

The two overproduced mutant proteins were purified and submitted to TSA. Unfortunately, despite several attempts using modified protocols for protein production and purification, a high fluorescence value was systematically observed at the onset of the TSA experiments and the curve of the first derivative of fluorescence emission relative to temperature revealed only the peak related to the MBP moiety. This probably indicates that the mutant LBD1056 moieties are not properly folded and tend to aggregate, resulting in dye binding even at low temperature. We, therefore, cannot conclude whether or not these conserved residues play a role in ligand binding by SO_1056.

Since the LBD of SO_1056 seems not to bind amino acids and contains an N residue instead of the key D residue present in the AA_motif of chemoreceptors detecting amino acids, we wondered whether this N residue could play a role for specific ligand binding in SO_1056. We, therefore, overproduced a mutated version of LBD1056, in which N170 was replaced by an aspartate. The overproduced protein was purified and submitted to TSA. As it was the case for its wild‐type counterpart, two peaks were observed for the MBP‐LBD1056_N170D_, one with Tm of 38.6 °C and the second with a Tm of 56.9 °C. When the MBP‐LBD1056_N170D_ protein was incubated in the presence of maltose, the Tm of the second peak was shifted to 62.3 °C, confirming that this peak corresponds to the MBP moiety (Table 1). It is noteworthy that the Tm of the mutated LBD1056 is higher than its wild‐type counterpart. When the MBP‐LBD1056_N170D_ protein was incubated in the presence of 0.1 and 1 mm malate, the Tm of LBD1056_N170D_ was up‐shifted of 0.9 and 2.4 °C, respectively (Table 1). When the experiment was performed with 0.1 and 1 mm nickel, the Tm of LBD1056_N170D_ was down‐shifted of 1.8 and 3.6 °C, respectively (Table 1). When the wild‐type version of MBP‐LBD1056 was used, the Tm of LBD1056 was up‐shifted of 12.8 °C in the presence of 1 mm malate and down‐shifted of 5.2 °C in the presence of 1 mm nickel (Fig. 2). These results suggest that the mutated version of LBD1056 is strongly affected for malate binding but only weakly for nickel binding, suggesting that N170 is involved in malate detection. It is noteworthy that none of the amino acids tested on this mutated version of MBP‐LBD1056 triggered a significant Tm shift of the LBD moiety, meaning that the replacement of N170 by D was not sufficient to convert LBD1056 from malate binding to amino acid binding.

To gather additional cues on ligand binding by SO_1056, we submitted the LBD1056 sequence to the alphafold software (Deepmind, London, UK). The structure model presented a long helix and two α/β pockets (Fig. 4B). This model has a high degree of confidence and is typical of the dCache fold found in various chemoreceptors including PctA. Interestingly, while the region of ligand binding in PctA is rather negatively charged, the corresponding region of LBD1056 is clearly positively charged (Fig. 4C). Consequently, this region of LBD1056 could indeed be a good candidate for binding the negatively charged l‐malic acid.

### Deletion of *so_1056* affects the chemotactic behaviour of *S. oneidensis* towards malate

**Fig. 5:**
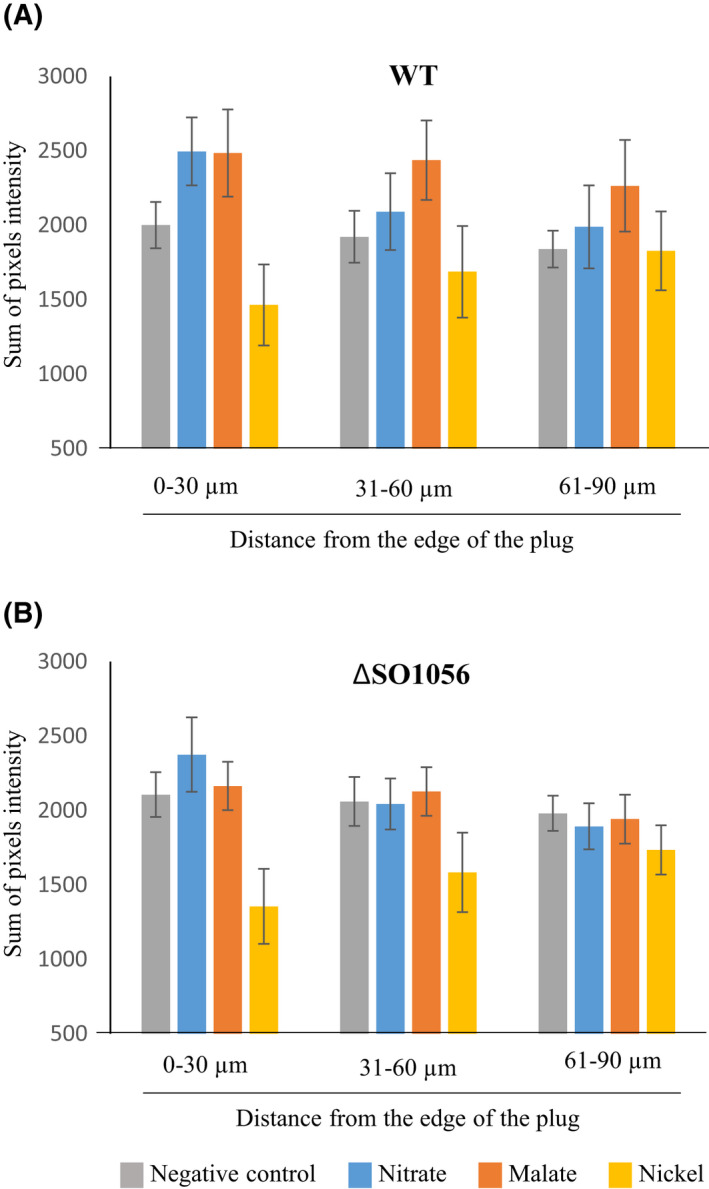
Chemotactic behaviour of *Shewanella oneidensis* cells towards various compounds. The chemotactic behaviours of the strains, wild‐type (WT, A) or deleted of the *so_1056* gene (ΔSO1056, B), towards a plug that contained either water (negative control), nitrate (50 mm), l‐malate (25 mm) or nickel sulfate (5 mm) were tested using an agarose‐in‐plug bridge assay. Photographs were taken at the edges of the plug after 22 min of incubation. Pixel intensities of each photograph, which are proportional to the number of cells around the plugs, were obtained using imagej. Graphs represent the sum of intensities of pixels located within 30 μm ranges along a 90 μm axis starting from the edge of the plug. Error bars represent the standard deviation of the mean (*n* = 20).

To determine whether SO_1056 detects l‐malate and nickel in *S. oneidensis*, we deleted the *so_1056* gene on the chromosome and tested the chemotactic behaviour of the resulting strain (ΔSO1056) towards malate and nickel using the agarose‐in‐plug bridge assay. In addition to the negative control, we included in our experiments an attractant (nitrate), which was previously shown to be detected indirectly via an energy tactic mechanism involving specific chemoreceptors [16]. As shown in Fig. 5A, when nitrate or malate was added to the plug, a higher sum of pixels, indicative of a higher number of cells, was observed at the closest range (0–30 μm) compared with the negative control for the wild‐type strain, confirming the attraction towards nitrate and malate. A similar phenomenon was also observed for malate at further ranges, while the response to nitrate was clearly lowered at those ranges. When the *so_1056* deleted strain was used, a higher number of cells was present at the 0–30 μm range when nitrate was added to the plug (Fig. 5B). In contrast, when malate was used, the sum of pixels was similar to that observed when no effector was added to the plug (negative control) whatever the distances from the plug (Fig. 5B). Therefore, attraction towards malate is strongly affected in the *so_1056* deleted strain. When nickel was added to the plug, the number of cells at the closest range was lowered compared with the negative control both for the wild‐type and the *so_1056* deleted strains, indicating that deletion of *so_1056* seems not to affect nickel repulsion in *S. oneidensis* (Fig. 5).

Altogether, these results suggest that the SO_1056 chemoreceptor plays a major role for the chemotactic response towards malate and probably only a minor one for that towards nickel.

## Discussion

We report the identification of an *S. oneidensis* chemoreceptor involved in both attraction and repulsion. Indeed, MCP SO_1056, when overexpressed, triggers an enhanced chemotactic response towards two attractants (chromate and malate) and away from two repellents (nickel and cobalt). Subsequently, we showed that signal detection is direct for chromate [8], malate and nickel (this study) and involves the LBD of SO_1056 which contains a dCache. Finally, we found that attraction towards malate is abolished in the *so_1056* deleted strain, suggesting a major involvement of SO_1056 in the chemotactic behaviour towards malate.

To the best of our knowledge, SO_1056 is the only described chemoreceptor responding to and directly binding chromate. Concerning malate, nickel and cobalt, chemoreceptors involved in their detection have been identified in several organisms. Strikingly, detection of malate by direct binding to chemoreceptor LBDs has been shown to involve diverse types of domains. Among the chemoreceptors described, PA2652 (*P. aeruginosa*) and Htc2 (*Halomonas titanicae*) detect malate via their sCache‐containing LBDs with K_*D*_ of 23 and 30.4 μm, respectively [28, 29]. For McpT (*Sinorhizobium meliloti*) and MCP2201 (*Comamonas testosteroni*), binding of malate occurs via their 4HB‐containing LBDs (K_*D*_ of 813 and 18.3 μm, respectively) [30, 31]. Malate direct binding has also been shown to occur via HBM‐containing LBDs, as in the case of the *Pseudomonas putida* McpS chemoreceptor with a K_*D*_ of 8.5 μm [32]. More recently, Tlp10 of *C. jejuni*, a chemoreceptor bearing a new kind of domain (DAHL—double all‐helical ligand‐binding domain), has been demonstrated to detect multiple classes of ligands, including malate [33]. Finally, dCache domains have also been found to be involved in malate detection. Although the chemoreceptor Tlp3 (*C. jejuni*) was first described to directly bind malate, a subsequent study using different binding tests proposed indirect detection of malate by this MCP [27, 34]. Another dCache‐containing chemoreceptor, TlpA of *Helicobacter pylori*, binds malate with a K_*D*_ of 46 μm but, strikingly, malate seems not to be a chemoeffector in this organism [35]. SO_1056 is, therefore, a clear example of a dCache‐containing chemoreceptor interacting directly with malate. The K_*D*_ measured for malate binding to SO_1056 (3.4 μm) is within the range of that in previous studies and agrees well with the observation that ligands bind with moderate affinity to chemoreceptors [36]. It is worth noting that, in addition to chemoreceptors, other families of proteins involved in signal transduction have also been shown to directly bind malate via their detection domain. For example, the histidine kinase DcuS from *E. coli* and the phosphatase RbsU from *Chlamydia trachomatis* have been proved to bind malate via sCache and dCache domains, respectively [37, 38]. Altogether, it seems that malate binding can occur via diverse domains, as previously noted [28]. A recent review listing the current knowledge on signal molecules and their interaction with diverse LBDs clearly established that this phenomenon is observed for several molecules and the authors proposed that these molecules could be of particular physiological relevance [36]. For example, amino acids, which are detected by a large diversity of domains, are of course indispensable for protein biosynthesis, but many of them can also serve as C‐ or N‐sources. In accordance with this assumption, malate could, therefore, have an important physiological role. Indeed, it has been found to be present in plant exudates and used by some soil bacteria as a signal to localize nutrient‐rich plant roots [39]. To date, we do not know the physiological role played by malate in *S. oneidensis*. It is probably not used as a C‐source but, as malate is excreted by marine phytoplankton, it could be used by *S. oneidensis* as a signal to localize nutrient‐rich zones in its environments.

Less information is available concerning nickel and cobalt detection by chemoreceptors. In *E. coli*, these molecules act as repellents [40] and their detection is mediated by the aspartate/maltose chemoreceptor Tar. Ni^2+^ detection by Tar has been found to be direct and involves the N‐terminal periplasmic domain of Tar [41, 42]. Nickel has also been found to be a repellent for *H. pylori*, but no specific chemoreceptor has been attributed to its detection [43]. Nickel and cobalt are reported to be recognized by about 10 different LBD families, but these LBDs are not present on chemoreceptors but rather on transcriptional regulators [36]. Therefore, Tar and SO_1056 are to date the only chemoreceptors reported to be involved in cobalt and nickel detection. In both cases, nickel is detected directly by the MCP, but the LBDs involved are different. Concerning cobalt, although weak binding to LBD1056 cannot be entirely excluded, indirect binding is more likely to occur. Although this is quite surprising because nickel and cobalt are metals of the same family, cobalt could be detected by a periplasmic binding protein (PBP). Indeed, the involvement of PBPs in ligand detection by chemoreceptors has been reported in several organisms. For example, inorganic phosphate is detected by two MCPs in *P. aeruginosa* and, for one of them, phosphate detection is indirect and involves the PstS periplasmic protein [44]. Chemotaxis mediated by PBP is also well known in *E. coli* and *Salmonella typhimurium*, especially for sugar detection [45, 46]. About 40 PBPs can be predicted from the *S. oneidensis* genome, and it will be interesting to decipher whether some of them are involved in chemotaxis by binding to specific chemoreceptors. Alternatively, direct detection of cobalt by SO_1056 could still be envisaged, but might involve a domain different from the LBD, such as the cytoplasmic region. Indeed, it was very recently shown that McpA from *B. subtilis* detects phenol, a repellent at high millimolar concentration, by its signalling domain rather than its sensing dCache‐containing domain [47].

Our results reinforce the idea that dCache domains are able to bind a large diversity of ligands. Although many of them have been proved to bind amino acids, some dCache‐containing chemoreceptors have been shown to bind other kinds of molecules, such as lactate, fumarate, malate, polyamines, histamine and benzoate derivatives [29, 35, 48, 49, 50]. Surprisingly, SO_1056 dCache shares similarities with the PctA chemoreceptor described to bind amino acids, while *S. oneidensis* does not show any chemotactic behaviour towards amino acids [20]. Therefore, experimental data are still crucial for identifying the ligands of a chemoreceptor, even though cues are provided by the increasing repertoire of ligand‐LBD interactions [36].

DCache domains present two pockets, one membrane‐distal and one membrane‐proximal. Most studies report ligand binding to the membrane‐distal pocket. Until recently, the only example involving a membrane‐proximal domain was that of the TlpC chemoreceptor detecting lactate [48]. A more recent study demonstrated that the dCache‐chemoreceptor TlpA of *H. pylori* is capable of binding seven ligands via both the membrane‐distal and proximal pockets [35]. Since SO_1056 dCache binds ligands of diverse nature, one attractive hypothesis would be that it does so by using its two pockets, one for example for malate binding and the other one for metal ion binding. Our first attempts to test this by using mutated versions of LBD1056 were only successful for the replacement of N170 by an aspartate and allowed us to conclude that N170 could be involved in malate binding. Nevertheless, further mutagenesis experiments will be necessary to unravel which pockets of LBD1056 are involved in the binding of its ligands.

In conclusion, we described here a dCache‐chemoreceptor involved in the detection of multiple ligands in *S. oneidensis*. Interestingly, these ligands differ not only in nature (dicarboxylic acid and metal ion) but also in the chemotactic effect they trigger (attraction and repulsion). We propose that this major chemoreceptor allows *S. oneidensis* to find nutrients or respiratory substrates and to escape deleterious environments.

## Materials and methods

### Strains and plasmids

In this work, we used *S. massilia*, *S. oneidensis* MR1‐R (deleted or not of *so_1056*) and *E. coli* C600 strains [23, 51]. The strains were routinely grown in lysogeny broth (LB) medium, at 28 °C for *Shewanella* strains and at 37 °C for *E. coli*. When necessary, ampicillin (50 μg·mL^−1^), chloramphenicol (25 μg·mL^−1^) or rifampicin (10 μg·mL^−1^) was added.

The pSO1056 plasmid corresponds to the pBAD33 vector, in which the entire *so_1056* gene was cloned [8]. The pMAL‐LBD‐1056 plasmid is derived from the pMAL‐c2X vector and contains the sequence coding for the LBD of SO_1056 (S29 to M278) fused to the sequence coding for MBP [8]. To construct the pMAL‐LBD‐1056W125A, pMAL‐LBD‐1056LLDS and pMAL‐LBD‐1056N170D plasmids, the pMAL‐LBD‐1056 plasmid was amplified by PCR using appropriate primers, following the protocol of the Q5 Site‐directed Mutagenesis kit (New England Biolabs, Ipswich, MA, USA). All constructs were checked by DNA sequencing using appropriate primers.

### Agarose‐in‐plug bridge assays

Microscope slide bridges were constructed as described previously [8, 17]. Briefly, a microscope slide bridge was constructed by placing two square coverslips on each side of a PETG slide. Four agarose plugs per slide were made by pipetting 5 μL of preheated 2% low‐melting agarose, prepared in distilled water, which contained the chemical to be tested onto the slide. Within a few seconds, a glass coverslip (24 × 60 mm) was placed over the slide and kept there by using a cyanoacrylate adhesive.

*Shewanella massilia* strain containing either the empty vector pBAD33 [52] or the pSO1056 plasmid was grown overnight on LB‐agar plates containing chloramphenicol (25 μg·mL^−1^) and arabinose (0.2%). *Shewanella oneidensis* (wild‐type and deleted of *so_1056*) were grown on LB‐agar plates. Cells were streaked, resuspended in liquid LB and then centrifuged for 10 min at 820 *g*. The cell pellets were gently resuspended in LM medium (0.2 g·L^−1^ yeast extract, 0.1 g·L^−1^ peptone, 10 mm HEPES (pH 7.4) and 10 mm NaHCO_3_) containing 20 mm lactate and 0.1% Tween‐20 to obtain an OD_600_ value of 0.25. After 30 min of static incubation at room temperature, 150 μL of cell suspension was introduced within the microscope slide bridge. Photographs were taken at the edges of the plugs after 20–40 min with a 4× phase contrast objective. Images were then converted from RGB to 16‐bit and pixels intensities, which are proportional to the number of cells located around the plugs, and were recorded using imagej [53]. For each image, profiles of pixels intensity located along 10 independent axis of 90 μm each starting from the edge of the plugs were determined and intensities of pixels located within 30 μm ranges along each axis were summed. Means and standard deviations of the sums calculated for each of the 10 axis were then determined.

### Expression and purification of MBP‐LBD1056 chimeric protein

The recombinant MBP‐tagged protein (MBP‐LBD1056) was produced from *E. coli* C600 strain containing the pMAL‐LBD‐1056 plasmid as previously described [8]. Briefly, the strain was grown aerobically in LB medium at 37 °C until an OD_600_ of 0.5 was reached. Expression was then induced with 0.3 mm of IPTG. After 3 h of growth at 28 °C, the cells were harvested and then resuspended in 20 mm Tris/HCl (pH = 7.4), 2 mm EDTA and 200 mm NaCl. Afterwards, they were lysed by a French press and centrifuged at 4 °C for 20 min at 15 557 *g*. The supernatant was filtered through a 0.45 μm membrane and loaded onto an amylose high‐flow resin (NEB). Thereafter, proteins were purified according to the manufacturer's protocol. Purified fractions (Fig. S1) were stocked at −80 °C with the addition of 10% glycerol. Their concentrations were determined either by the Bradford assay (Bio‐Rad, Hercules, CA, USA) or by OD_280_ measurement.

The mutated versions of MBP‐LBD1056 (MBP fused to LBD1056 with W125 replaced by A, to LBD1056 with R123P124W125Y126 replaced by LLDS and to LBD1056 with N170 replaced by D were produced from *E. coli* C600 strain containing plasmid pMAL‐LBD‐1056W125A, pMAL‐LBD‐1056LLDS or pMAL‐LBD‐1056N170D, respectively, using the same protocol.

### Thermal shift assays (TSAs)

Purified proteins were first loaded onto an NAP‐5 desalting column (GE Healthcare, Chicago, IL, USA) and recovered in 20 mm Tris/HCl (pH 7.4) containing 100 mm NaCl and 10% glycerol. TSAs were performed in a total volume of 20 μL, as described previously [8, 54]. In summary, MBP‐LBD1056 (6.2 μm) was incubated in the presence of 10× SYPRO Orange (Sigma‐Aldrich, St. Louis, MO, USA) with or without l‐malate, nickel chloride and cobalt chloride (100 μm and 1 mm). As controls, we used fumarate, molybdate and tungstate (1 mm). Tests were also performed with l‐Methionine, l‐Tryptophane and l‐Serine (100 μm, 1 mm and 4 mm). Samples were then heated from 10 to 90 °C at a scan rate of 0.5 °C per 30 s using a BioRad CFX96 Touch Real‐Time PCR instrument. Protein unfolding curves were monitored by detecting changes in SYPRO Orange fluorescence. Melting temperatures were determined using the first derivative values of raw fluorescence data using Bio‐Rad cfx manager 3.1 software.

### Isothermal titration calorimetry (ITC)

The purified MBP‐LBD1056 protein was first dialysed against buffer (20 mm Tris/HCl pH = 7.4; 200 mm NaCl) three times for 1 h. ITC experiments were performed using a MicroCal PEAQ‐ITC calorimeter (Malvern, UK) at 25 °C with the following settings: 2 μL injection volume; 19 injections; 750 r.p.m. stir speed. The sample cell was loaded with 300 μL of MBP‐LBD1056 (15 μm) in dialysis buffer. MBP‐LBD1056 was then titrated with the ligands diluted in dialysis buffer. A control was made for each experiment using dialysis buffer only in the cell and ligand in the syringe. Experimental data were fitted using Hill equation and sigmaplot software (Inpixon, Palo Alto, CA, USA). The experiments were performed in duplicate using independently purified MBP‐LBD1056; one representative result was shown.

### Homology search and structure prediction

The amino acid sequence of the SO_1056 protein between Ser29 and Met278 was uploaded to the Smart BLAST server (https://blast.ncbi.nlm.nih.gov/smartblast/). Structure prediction was performed using the software alphafold and the same sequence as a query [55]. The electrostatic surface was calculated with the default coulombic command in chimerax (https://www.cgl.ucsf.edu/chimerax/index.html).

## Conflict of interest

The authors declare no conflict of interest.

## Author contributions

Conceptualization, AB, J‐PP, AAC, H‐PF, M‐TG‐O, VM and CJ‐C; methodology, AB, J‐PP, AAC, H‐PF, M‐TG‐O, VM and CJ‐C; validation, AB, J‐PP, AAC, H‐PF, M‐TG‐O, VM and CJ‐C; investigation, AB, J‐PP, AAC, H‐PF, M‐TG‐O, VM and CJ‐C; writing—original draft preparation, AB, J‐PP, VM and CJ‐C; writing—review and editing, AB, J‐PP, H‐PF, M‐TG‐O, VM and CJ‐C; supervision, VM and CJ‐C; funding acquisition, M‐TG‐O, VM and CJ‐C. All authors have read and agreed to the published version of the manuscript.

## Supporting information


**Fig. S1.** Sodium dodecyl sulfate‐polyacrylamide gel electrophoresis (SDS/PAGE) and protein staining.

## Data Availability

The authors declare that all relevant data supporting the findings of the study are available in this article and the Supporting Information section, or from the corresponding author upon reasonable request.
