# Supplementary material for: Multiple detection of both attractants and repellents by the dCache‐chemoreceptor SO_1056 of Shewanella oneidensis
Source: FEBS J. 2022 Jun 24;289(21):6752–66. doi: 10.1111/febs.16548 (PMC9796306; doi:10.1111/febs.16548)
Supplement: Supplementary file 1 — Fig. S1. Sodium dodecyl sulfate‐polyacrylamide gel electrophoresis (SDS/PAGE) and protein staining. [file FEBS-289-6752-s001.pdf]

Supplemental informations

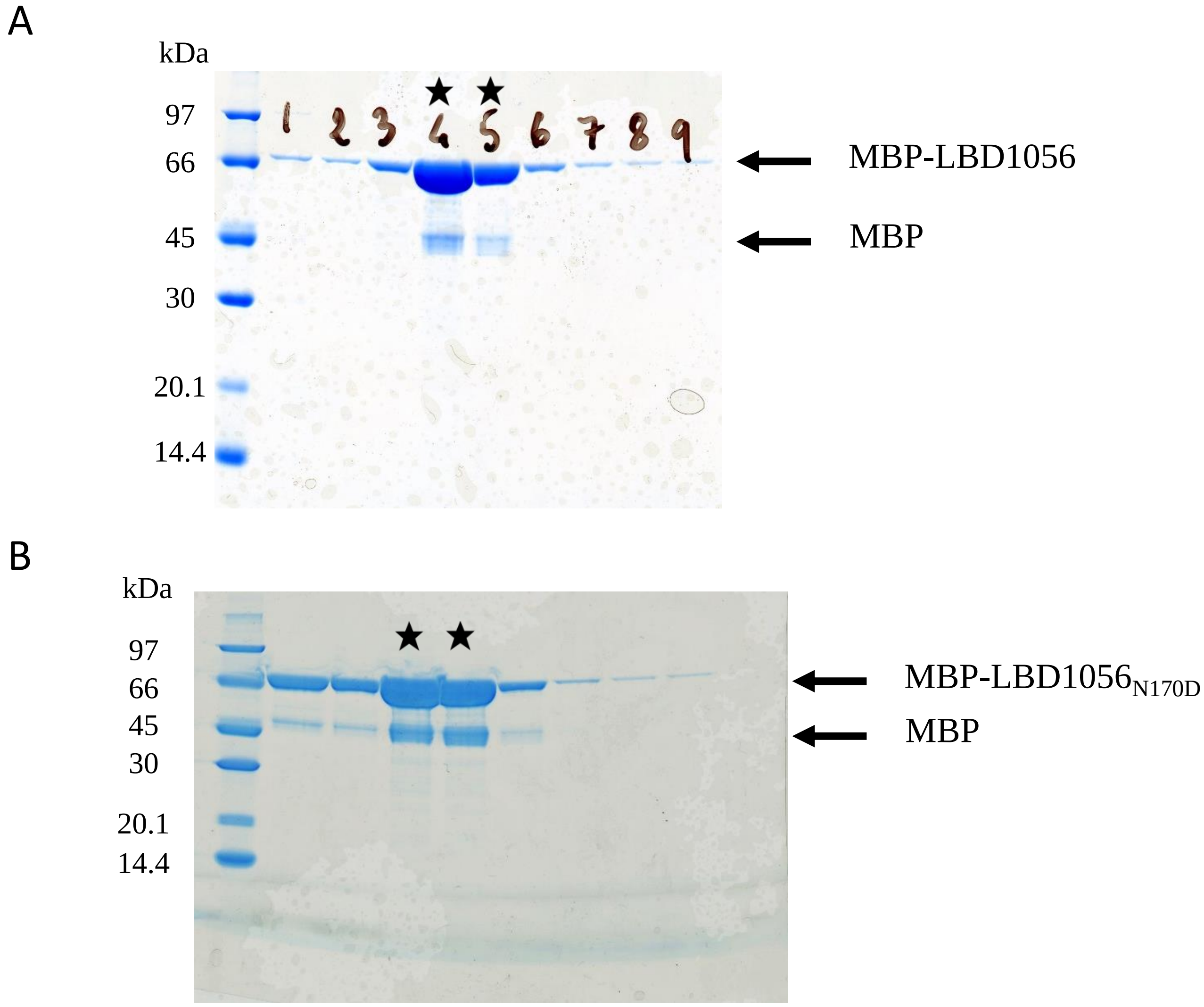

Figure S1. Sodium dodecyl sulfate-polyacrylamide gel electrophoresis (SDS-PAGE) and protein staining. 20 µL of each purified fractions of MBP-LBD1056 (A) and MBP-LBD1056$_{N170D}$ (B) were mixed with 10 µL of TSTD (133 mM Tris/HCl, pH 8.8, 3.3 mM EDTA, 0.7 M sucrose, 0.07% bromophenol blue, 6% SDS, 0.1M DTT and 1.7% β-mercaptoethanol) and denatured by heating at 95 °C for 5 min. Samples were then loaded onto a 4-20% gradient polyacrylamide SDS-PAGE gel (Shanghai WSHT Biotechnology Inc. China). The migration was processed for 1h at 120V and proteins visualization was done by gel staining with InstantBlue™ (Sigma-aldrich, Saint-Louis, USA). Fractions number tagged with a (★) were used for Thermal Shift Assays (TSAs; A and B) and Isothermal Titration Calorimetry (ITC; A). The picture is representative of what was obtained in several purification experiments.
